# Substance use and sociodemographic correlates among adolescents in a low-income sub Saharan setting

**DOI:** 10.5249/jivr.v12i1.1195

**Published:** 2020-01

**Authors:** Anne Abio, Jurdas Sezirahiga, Laura E. Davis, Michael L. Wilson

**Affiliations:** ^*a*^Injury Epidemiology and Prevention Research Group, Turku Brain Injury Center, Division of Clinical Neurosciences, Turku University Hospital and University of Turku, Finland.; ^*b*^PeerCorps Trust Fund, 352/64 Makunganya Street, Co-Architecture Building, 4th Floor, Dar es Salaam, Tanzania.; ^*c*^Injury Epidemiology and Prevention Research Group, Turku Brain Injury Center, Division of Clinical Neurosciences, Turku University Hospital and University of Turku, Finland.; ^*d*^Heidelberg Institute of Global Health, University of Heidelberg, Germany.

**Keywords:** Substance use, Adolescents, Epidemiology, Sub Saharan Africa

## Abstract

**Background:**

The present study investigated the social and behavioral correlates of substance use, specifically bang or cocaine or similar drugs among a school-based sample of adolescents in a low-income urban setting.

**Methods:**

The study utilized data on 2,176 school-attending adolescents aged 11-16 years in Dar es Sa-laam (DES) to examine social and behavioral correlates for lifetime substance use. The correlates under investigation included, demographic - age and gender; social - poverty, parent-adolescent relationship; behavioral – truancy, aggressive behavior, injury risk; psychological- depression and suicide ideation. Bivariate and multivariate analyses were then carried out on several variables identified from the 2006 Tanzanian Global School-based Health Survey (GSHS) questionnaire.

**Results:**

Approximately seven percent of school-attending adolescents (n=144) reported having used an illicit substance at least once during their lifetime. After adding significantly associated covari-ates into a logistic regression model, we found that only truancy [OR= 2.29 (CI=1.07– 4.90)], suicide ideation [OR=4.36 (2.32 – 8.19)] and parents who had checked their adolescents' homework (OR=0.56 (CI=0.32 – 1.00)] were significantly associated with reported substance use.

**Conclusions:**

Our results suggest that health promotion programs should simultaneously target multiple factors associated with substance use behaviors among school-attending adolescents in Dar es Salaam. They should take into account the range of psychosocial characteristics of school-attending adolescents which may be impacted by or result from substance use.

## Introduction

Substance use among adolescents is a growing problem of public health importance and research suggests that substance use behaviors are on the rise.^1,2^ In their examination of contributors to the global burden of disease, Rehm et al. (2006) documented that substance use behaviors accounted for approximately 4% of the disability adjusted life years.^3^ Substance use differs by age and gender. ^4,5^

Substance use behaviors among adolescents have been found to be associated with unintentional injuries, violent behavior, weapon carrying, sexual risk taking, drink-driving, overdose, self-harm, toxicity and death.^6-10^ Furthermore, it has also been associated with socioeconomic status, suicidal ideation, truancy, depression and having poor relationships with parents in several studies.^11-14^ Adolescence is a transition period to adulthood and substance use during this important phase could potentially lead to dependence depending on access and availability of illicit drugs thus preventive measures are important where deemed necessary.^15^

Regrettably, most research on substance use patterns, utilizes data derived from mainly high-income country settings. Thus the literature remains silent on the extent to which harmful substance use patterns have been examined in low- and middle-income country settings. Furthermore, in low- and middle-income settings which do report on substance use patterns among their populations, few if any provide data that has been disaggregated by age^13^ (Peden et al., 2008). 

In Tanzania, substance use is on the rise and has been associated with the spread of HIV especially through sharing injections especially in the densely populated cities like Dar es Salaam. A study found cannabis use estimated at 0.8%, more so among younger people in 2003 in Dar es Salaam.^16^ Within specific populations, a psychiatric patient population had a prevalence of 29% cannabis use in Mwanza.^17^ In another study conducted in Mwanza in 2014, shared housing and cohabiting was found to be associated with injection drug use among drug users.^18^

The aim of the present study was to explore the sociodemographic factors associated with substance use in a low-income urban sub-Saharan setting.

## Methods 

**Setting**

The present study employs the use of data derived from Dar es Salaam (DES) in the United Republic of Tanzania. Tanzania has a population of approximately 50 million people and roughly 70% live in rural areas as of 2016.^19^ Forty-four percent of the population are below the age of 14 years. Dar es Salaam city is a large commercial center and former capital of Tanzania located on the East African coast facing the Indian Ocean. Its population of approximately 5.5 million, has the highest annual growth rate in the country at 5.6%.^20^

**Sample**

Our data are drawn from the 2006 Tanzanian Global School-based Student Health Survey (GSHS), a self-administered questionnaire which collects information on risk and protective factors for school-attending adolescents in 43 countries. In Tanzania, data were collected via a two-stage cluster sampling procedure representative of all secondary schools in DES. At stage one, schools were selected with a probability being proportional to the school's enrollment size. At stage two, classes were randomly selected with all students in selected classes being eligible to participate. The school response rate was 100% with the overall student response rate being 87%. A total of 2,176 students aged 11-16 years (approximately 3.5% of the sample responded to being aged 16 years or older) participated in the survey. We excluded 22 participants who did not have complete data resulting in a final sample of 2,154 (52% were female). At the time of data collection, the study was approved by the Ministry of Health and Social Welfare.

**Measurements**

We defined substance use using one question provided in the survey questionnaire: “During your life, how many times have you used drugs such as bang or cocaine?”. The responses were “0 times”, “1 or 2 times”, “3 to 9 times” and “10 or more times”. We dichotomized these responses into “0 times” (reference category) and “1 or more times” so that a logistic regression model could be used to facilitate the examination of the effect of demographic exposures on drug use as the dependent variable. Substance use differs by age and gender.^2,4^ In addition, several studies ^11-14,21^ have found that substance use among adolescents to be associated with socioeconomic status, increased risk for injury and violence, suicidal ideation, truancy, depression and having poor relationships with parents. 

These associations in the present sample were explored using the following questions from the GSHS:

Poverty was examined using the question: "During the past 30 days, how often did you go hungry because there was not enough food in your home?". The responses were "never", "rarely", "sometimes", "most of the time", or "always" and were dichotomized into "sometimes/most of the time/always" against “never/rarely”. This was because never or rarely going hungry implies the family is able to provide the basic necessities for example food as opposed to sometimes or mostly failing to provide basic needs in a home. Truancy was explored using the question: "During the past 30 days, on how many days did you miss classes or school without permission?" The responses were "0 days", "1 to 2 days", "3 to 5 days", "6 to 9 days", and "10 or more days". Students were considered truant if they missed more than 3 days of school within the 30 days prior to the survey as missing 1 or 2 days may be reasonable.

The parent-adolescent relationship was examined using: "During the past 30 days how often did your parents or guardians check to see if your homework was done?"; "During the past 30 days, how often did your parents or guardians understand your problems and worries?"; and "During the past 30 days, how often did your parents or guardians really know what you were doing with your free time?". The responses to each were "never", "rarely", "sometimes", "most of the time", or "always". For each of these we dichotomized the responses into "most of the time/sometimes/always" and “rarely/never”. This is because if parents/guardians never or rarely check homework and/or know what their children are doing during their free time may imply a lack of parent/child bond as opposed to if they are mostly aware of the children’s activities. Aggressive behavior was examined using the question: “During the past 12 months, how many times were you in a physical fight?”. The responses were “0 times”, “1 time”, “2 or 3 times”, “4 or 5 times”, “6 or 7 times”, “8 or 9 times”, “10 or 11 times”, “12 or more times”. We dichotomized the responses into “0 times” and “1 or more times” because any number of times adolescents may have been in a fight be indicative of aggression.

Injury risk was examined using the question: “During the past 12 months, how many times were you seriously injured?”. The responses were “0 times”, “1 time”, “2 or 3 times”, “4 or 5 times”, “6 or 7 times”, “8 or 9 times”, “10 or 11 times”, “12 or more times”. We dichotomized the responses into “0 times” and “1 or more times” to indicate any number of times one may have been injured. Suicide ideation was explored using the question: “During the past 12 months, did you ever seriously consider attempting suicide?”. The responses were (Yes/No) and used as provided. Finally, we explored depression using the question: "During the past 12 months, did you ever feel so sad or hopeless almost every day for two weeks or more in a row that you stopped doing your usual activities?” (Yes/No). Data was collected for the mentioned question based on the 2 categories and used as provided.

**Statistical analysis**

We first conducted bivariate analyses in which we tested for associations with substance use behavior. We used Pearson's chi-square for categorical variables and the t-test for continuous variables (age). Variables were introduced in the multivariate regression model if significant in the bivariate analysis at p<0.05. All sociodemographic factors were included in the final regression model along with the family, interpersonal and mental health covariates which were found to be significant at the bivariate level. The sociodemographic factors were included because they are very frequently associated with situational and environmental risk factors (i.e. poverty status) and are thus controlled for.

Logistic regression was used to examine associations with each category compared with controls while adjusting for covariates. The measures of association for the regression model were reported as odds ratios (OR) with confidence intervals being computed at the 95% level. All analyses were conducted using IBM SPSS Statistics for Windows version 19.

## Results

Approximately seven percent of school-attending adolescents (n=144) reported having used a substance at least once in their lifetime from the survey. One percent reported having used substances three or more times. 

In the unadjusted analyses (Table 1), we found no significant associations between substance use and age, gender or socioeconomic status. Neither were having understanding parents or parents who were knowledgeable about their adolescents' free time activities significantly associated with substance use behavior. However, we found that school-attending adolescents who had reported substance use, were more likely to have been truant, been in a physical fight and suffered from a serious injury within the recall period. We also found higher rates of suicidal ideation and depression among those who reported substance use. Having parents who checked homework was significantly associated with adolescent reported substance use. 

**Table 1:** Bivariate analyses of lifetime illicit substance use among school-attending adolescents in Dar es Salaam, Tanzania (2006).

Variable	No lifetime illicit substance use – Percent (Number)	Lifetime illicit substance use, at least once – Percent (Number)	P-value
Mean age (SD)	13 (1.3)	12.8 (1.4)	0.175
Gender (male)	95.0% (951)	5.0% (50)	0.096
Poverty (yes)	94.7% (215)	5.3% (12)	0.366
Truancy (yes)	88.4% (152)	11.6% (20)	P<0.001
Been in a physical fight (yes)	94.5% (797)	5.5% (46)	0.025
Considered suicide (yes)	87.3% (200)	12.7% (29)	P<0.001
Depression (yes)	93% (451)	7% (34)	P<0.001
Seriously injured (yes)	94.2% (647)	5.8% (40)	0.002
Parents checked homework (yes)	97% (1343)	3% (41)	P<0.001
Understanding parents (yes)	96.5% (937)	3.5% (34)	0.083
Parents knowledgeable about free time (yes)	96.5% (1011)	3.5% (37)	0.102

Reference category: Negative response to the respective questions. The results are reported as proportions (%) and the total counts for all variables except for age for which the standard deviation is reported.

After adding significantly associated covariates into a logistic regression model (Table 2), we found that only truancy [OR= 2.29 (CI=1.07 – 4.90)], suicide ideation [OR=4.36 (2.32 – 8.19)] and parents who check their adolescents' homework (OR=0.56 (CI=0.32 – 1.00)] were significantly associated with reported substance use. 

**Table 2:** Multivariate analyses of lifetime illicit substance use among school-attending adolescents in Dar es Salaam, Tanzania (2006).

Variable	Lifetime illicit substance use (OR)	CI	P-value
Age	1.06	0.85 – 1.31	0.614
Gender	1.22	0.67 – 2.21	0.522
Poverty	0.52	0.18 – 1.50	0.225
Truancy	2.29	1.07 – 4.90	0.034
Been in a physical fight	1.05	0.57 – 1.92	0.889
Considered suicide	4.36	2.32 – 8.19	<0.001
Depression	1.63	0.88 – 3.02	0.119
Seriously injured	1.48	0.81 – 2.71	0.201
Parents checked homework	0.56	0.32 – 1.00	0.049

All variables adjusted for all covariates shown

## Discussion

The present study explored several correlates for substance use among school-attending adolescents in a low-income sub Saharan setting. After controlling for covariates in a logistic regression model, substance use was significantly associated with truancy, suicide ideation and adolescents whose parents checked their homework. Despite indications in the literature,^14^ we were not able to replicate associations between substance use and socioeconomic status, particularly low socioeconomic status after controlling for covariates. One hypothesis may be related to the lack of variability in the present sample. In addition, a lack of quantitative elements which could more adequately capture differences in relative economic well-being were not available for analysis. We were also unable to replicate findings concerning differences by gender and age.^22,23^ The prevalence of substance use among girls has been increasing globally, especially in high income countries.^24,25^ Dar es Salaam, an urban setting where the survey was conducted could be an area reflecting the reducing gender gap due to gender roles and urbanization.

While the risk of physical interpersonal violence and injury have been shown to increase with substance use, ^26^ our results were not demonstrative of this effect. Depression has been linked to substance use behaviors in several settings,^27^ including those in the sub-Saharan region. ^28^ As the prevalence of substance use among the sample was low, especially for more than one-time use, it is plausible that most substance use was experimental in origin. Thus, it is unlikely to be demonstrative of other behavioral risk patterns that have been associated in the literature with substance abuse.^29^

The main findings in this study include statistically significant associations with suicide ideation, truancy, and parent involvement in homework tasks, as observed in other studies. Parental support and supervision has been found to be protective of substance use among adolescents but not in all studies. Substance use was significantly associated with missing school among adolescents although it was found to be protective in Iraq with cannabis use. Suicide ideation tends to be associated with other mental disorders but also increase the risk of substance use.^30^ We hypothesize that the substance use in this study, may derive from attempts to cope with psychological and social pressures during adolescents.^31^ This may be the case especially for those who reported using substances more than once during their lifetime. Similarly, substance use in previous research has been linked with suicide ideation as a potential coping mechanism related to poor mental health. ^21^

**Strengths and Limitations**

This study provides important insight into substance use used among a rarely studied segment of the population in a low-income sub-Saharan setting. To our knowledge, it is the only study to have examined substance use patterns among school-attending adolescents in Tanzania. 

Despite this novelty, the results presented here must be viewed in light of their limitations. First, the study sample is representative only of adolescents that attended school in DES. Thus, while representative of school attending adolescents, the study remains silent on substance use patterns among adolescents who were absent on the day of the survey or who do not attend school at all. The substance use among the adolescents not attending school or school dropouts could be higher due to varying environment factors; however, initiation of drug use may also lead to missing school and/or eventually dropping out for those still in school. ^32-34^ Adolescents out of school may not be adequately supervised by their parents and thus be increasingly predisposed to using illicit substances.^35^ Secondly, although this survey was designed to be administered cross-culturally, there may have existed ambiguity in how questions were interpreted by respondents. For example, questions concerning mental health items such as depression, may manifest themselves differently cross culturally,^36^ and as such these symptoms may not be accurately captured when using Western measurement scales. The inclusion of information on family and peers would have strengthened the study. Thirdly, substance use, which was the primary variable under investigation and the correlates were measured based on a single question items, and no validation was done. This may explain a lack of variability and measurement error in the results. Finally, the survey respondents encompassed adolescents who responded affirmatively to being aged “16 years or older”. It was not possible to know whether the “or older” meant those who were 16 years of age and a few months or significantly older then their peers. While important to point out, the authors do not expect this to have had a significant impact on the responses or the conclusions reached subsequent to the analyses.

## Conclusion

This study identified social and behavioral factors that increased the risk and protected against illicit substance use among adolescents in Dar es Salaam. Our results suggest that health promotion programs should simultaneously target multiple factors associated with substance use behaviors among school-attending adolescents in DES. They should take into account the range of psychosocial characteristics of school-attending adolescents which may be impacted or be a result of substance use. Further research should seek to make use of culturally appropriate and validated questionnaires to allow for greater accuracy in examining parent child relationships and mental health.

**Acknowledgments**

The authors would like to thank the participants and school officials who contributed their responses during data collection. The World Health Organization is appreciated for making the GSHS data publicly available.

Author AA was funded by the EDCTP/TDR Clinical Research and Development Fellowship Program, World Health Organization, Geneva, Switzerland; the John Harvey Lowery Foundation, USA; and the University of Turku Joint Research Grant, Finland. Author MLW was funded by a grant from the Alexander von Humboldt-Stiftung, Bonn, Germany. Dimitrious Pilalas, M.D. is acknowledged for his assistance with the statistical analysis. 
